# Perioperative blood loss reduction using a sterile exsanguination tourniquet for orthopedic femoral-related surgeries in children: a randomized controlled study

**DOI:** 10.1186/s13018-023-04046-3

**Published:** 2023-08-08

**Authors:** Terapat Rattanathanya, Nath Adulkasem, Jidapa Wongcharoenwatana, Thanase Ariyawatkul, Chatupon Chotigavanichaya, Perajit Eamsobhana

**Affiliations:** Department of Orthopedic Surgery, Faculty of Medicine, Siriraj Hospital, Mahidol University, 2 Prannok Road, Bangkok Noi, Bangkok, 10700 Thailand

**Keywords:** Sterile elastic exsanguination tourniquet, Femur, Blood loss, Surgery, Pediatrics

## Abstract

**Objectives:**

The sterile exsanguination tourniquet (SET) could be an alternative for providing bloodless surgeries in orthopedic femoral-related surgeries in pediatric patients where the standard pneumatic tourniquet would not be feasible. This randomized-controlled study aimed to evaluate the efficacy of SET in decreasing total perioperative blood loss and blood transfusion.

**Methods:**

We conducted an unplanned interim analysis of data from a randomized-controlled trial. At the time of the analysis, 31 pediatric patients had been randomly assigned to undergo surgery with the SET application (the SET group, 15 patients) and without the SET application (the control group, 16 patients). An intention-to-treat analysis was performed to evaluate the total perioperative blood loss, postoperative blood transfusion, estimated intraoperative blood loss, total drainage volume, postoperative hemoglobin level, and operative time according to the significance level adjusted for multiplicity (*p* < 0.029).

**Results:**

There was a borderline statistically significant lower body weight-adjusted TBL in the SET group (SET = 14.1 (7.7, 16.9) ml/kg vs. control 18.3 (14.8, 37.2) ml/kg, *p*-value = 0.027). The body weight-adjusted transfusion volume was statistically significantly greater in the control group (SET = 0.0 (0.0, 0.0) ml/kg vs. control = 2.1 (0.0, 9.7) ml/kg, *p* = 0.017). Body weight-adjusted estimated intraoperative blood loss was significantly lower in the SET group (SET = 0.8 (0.2, 3.5) ml/kg vs. control = 5.6 (3.4, 21.5) ml/kg, *p* < 0.001). In addition, the operative time was lower in the SET group with borderline statistical significance (SET = 105 (85.0, 125.0) vs. control = 130 (101.3, 167.5), *p* =  0.039).

**Conclusion:**

Utilization of a sterile exsanguination tourniquet (SET) significantly reduced an estimated intraoperative blood loss while preventing the need for blood transfusion after pediatric orthopedic femoral-related surgeries.

*Trial registration* TCTR20220412003.

## Background

Perioperative blood loss prevention for femoral orthopedics surgery in the pediatric population is crucial since the children have less total blood volume [1]. A small amount of blood loss could significantly reduce Hb levels in pediatric patients [2]. Accordingly, the pneumatic tourniquet is widely used in orthopedic procedures to facilitate the bloodless surgical field and minimize perioperative blood loss [3]. Although previous studies highlighted the efficacy and safety of this device, the pneumatic tourniquet cannot be used when the procedure requires a surgical visualization adjacent to the application site [4]. For example, utilizing the standard pneumatic tourniquet in pediatric femoral-related surgery might be inappropriate. The limited application area and the child’s thigh geometry (short, tapered, conical shape) are susceptible to the distal sliding of the tourniquet during surgery, resulting in the loss of tourniquet compression while breaching of surgical field sterility [5]. Hence, femoral-related surgeries at the mid to proximal thigh are inappropriate for the standard pneumatic tourniquet (e.g., diaphyseal femoral fracture).

The sterile exsanguination tourniquet (SET) has been used to create a bloodless surgical field for various extremities surgeries [6]. SET is an elastic sterile stockinet device consisting of an elastic silicone ring, a stockinette, and straps with handles. This device was designed to remove the blood from the operated limb (exsanguination), maintain an arterial flow occlusion during the surgery, and provide a sterile bloodless surgical field [7]. SET can be applied as proximal as the groin level, accommodating the surgeries located distally. In addition, SET requires only a small area of application equal to the width of the silicone ring, which can be beneficial when the application area is limited (e.g., pediatric extremities).

Regarding the limitations of the standard pneumatic tourniquet, we hypothesize that SET could be an effective alternative for restricting perioperative blood loss and blood transfusion in orthopedic femoral-related surgeries in pediatric patients. This randomized-controlled study aimed to evaluate the efficacy of SET in terms of total perioperative blood loss and blood transfusion control in pediatric femoral-related surgeries. In addition, estimated intraoperative blood loss, total postoperative drained volume, and operative duration were also evaluated.

## Materials and methods

### Study design, randomization, and outcomes

We conducted a randomized-controlled trial to identify the efficacy of SET in reducing perioperative blood loss for pediatric femoral surgery. We included pediatric patients aged 3–18 who were scheduled for elective femoral-related surgery in which the standard pneumatic tourniquet was not appropriate due to the surgical field obstruction (e.g., diaphyseal femoral fracture and distal femoral derotation osteotomy with plate osteosynthesis). These procedures are ideal candidates for a SET application. Although SET can be applied as high as on the upper thigh, it can only be used for surgical interventions located below the application area. Therefore, patients scheduled for proximal femoral surgeries (e.g., proximal femoral fracture and femoral varus osteotomy) were not included. Exclusion criteria included patients with peripheral vascular disease, a history of deep vein thrombosis, a history of bone infection and malignancy, bleeding disorders, and any associated injuries affecting further blood loss.

After receiving informed consent, block-of-4 randomization was carried out using a computer-generated randomization sequence to allocate patients into two treatment arms equally; the treatment arm used SET, and the control arm didn’t use SET during the operation. Allocation was concealed using sequentially numbered, opaque, sealed envelopes (SNOSE) prior to making the incision. Treatment allocation was blinded to the patients and outcome accessor throughout the trial period.

Hemoglobin level (Hb) was recorded at postoperative 24 and 72 h. The primary outcomes were perioperative total blood loss (TBL) and transfusion rate at 72 h after surgery. TBL was calculated based on the formula provided by Tzazairis et al. [8]. Transfusion was performed if the postoperative Hb was less than 7 g/dl according to the Joint United Kingdom Blood Transfusion and Tissue Transplantation Services Professional Advisory Committee (JPAC) guidelines. Secondary outcomes included estimated intraoperative blood loss assessed by an attended anesthesiologist, total drained volume 24 h after surgery, and operative time. All volume measurements were presented in body weight-adjusted format (ml/kg) to improve clinical correlation.

The institutional review board (IRB) and departmental data safety monitoring board (DSMB) approved and monitored this study.

### Intervention

**Fig. 1:**
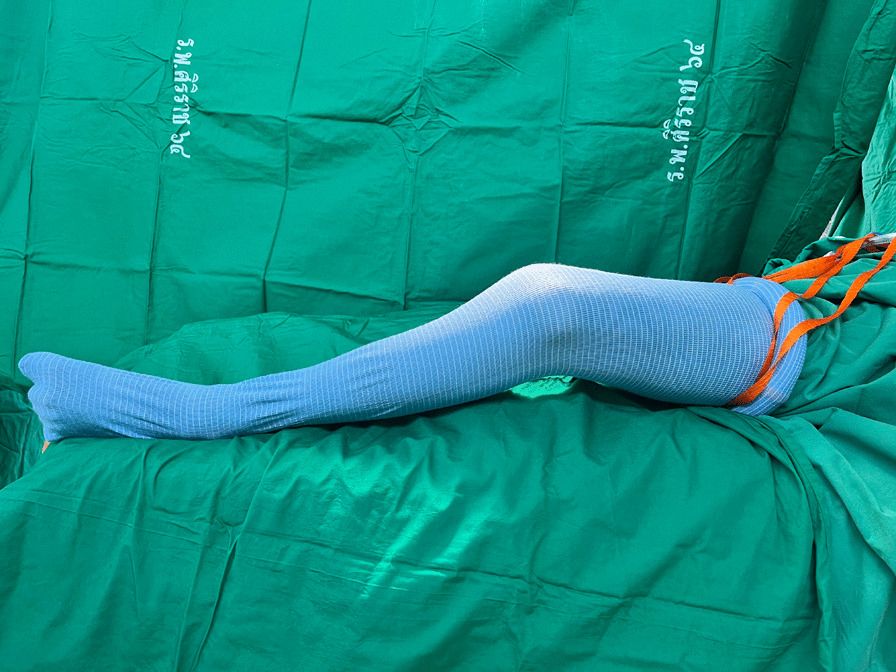
A sterile exsanguination tourniquet (HemaClear®, OHK Medical Devices, Inc., Grandville, MI, USA) was applied to the patient’s thigh

All patients in the SET arm were measured for their thigh circumference to select the appropriate tourniquet size before skin preparation and sterile surgical draping. A sterile exsanguination tourniquet (HemaClear^®^, OHK Medical Devices, Inc., Grandville, MI, USA) was applied on the patient’s thigh proximal to the planned surgical incision area to remove blood, arterial flow occlusion, and automatic application of a sterile stockinette (Fig. 1). The drainage was placed to collect the blood from the surgical site in both treatment arms. The SET was removed after finishing the surgery.

### Sample size

The sample size was calculated using the blood loss reduction in the femoral varus derotational osteotomy reported in Tzazairis et al. study [8]. As a result, the two-independent mean testing formula indicated that 20 patients for each treatment arm were sufficient for determining the efficacy of SET for femoral-related surgeries regarding *α* < 0.05, *β* < 0.20, and a dropout rate of 20%.

### Statistical analysis

All analyzes were performed using SPSS 18.0 (SPSS Inc., Chicago, IL, USA). According to data distribution, continuous data were presented with mean ± SD or median and interquartile ranges (IQR). Categorical data were presented with counts and percentages. Differences between treatment arms were analyzed using an independent *t*-test, Mann–Whitney *U* test, and Fisher’s exact probability test according to data nature and distribution. After the outcomes were collected for 75%, the DSMB expressed concern that the outcomes were markedly different between treatment arms and recommended an unplanned interim analysis of transfusion rate and TBL. Accordingly, we conducted an intention-to-treat analysis with an adjusted significance level of *p* < 0.029 for multiplicity according to Pocock’s approach [9, 10].

## Results

**Fig. 2:**
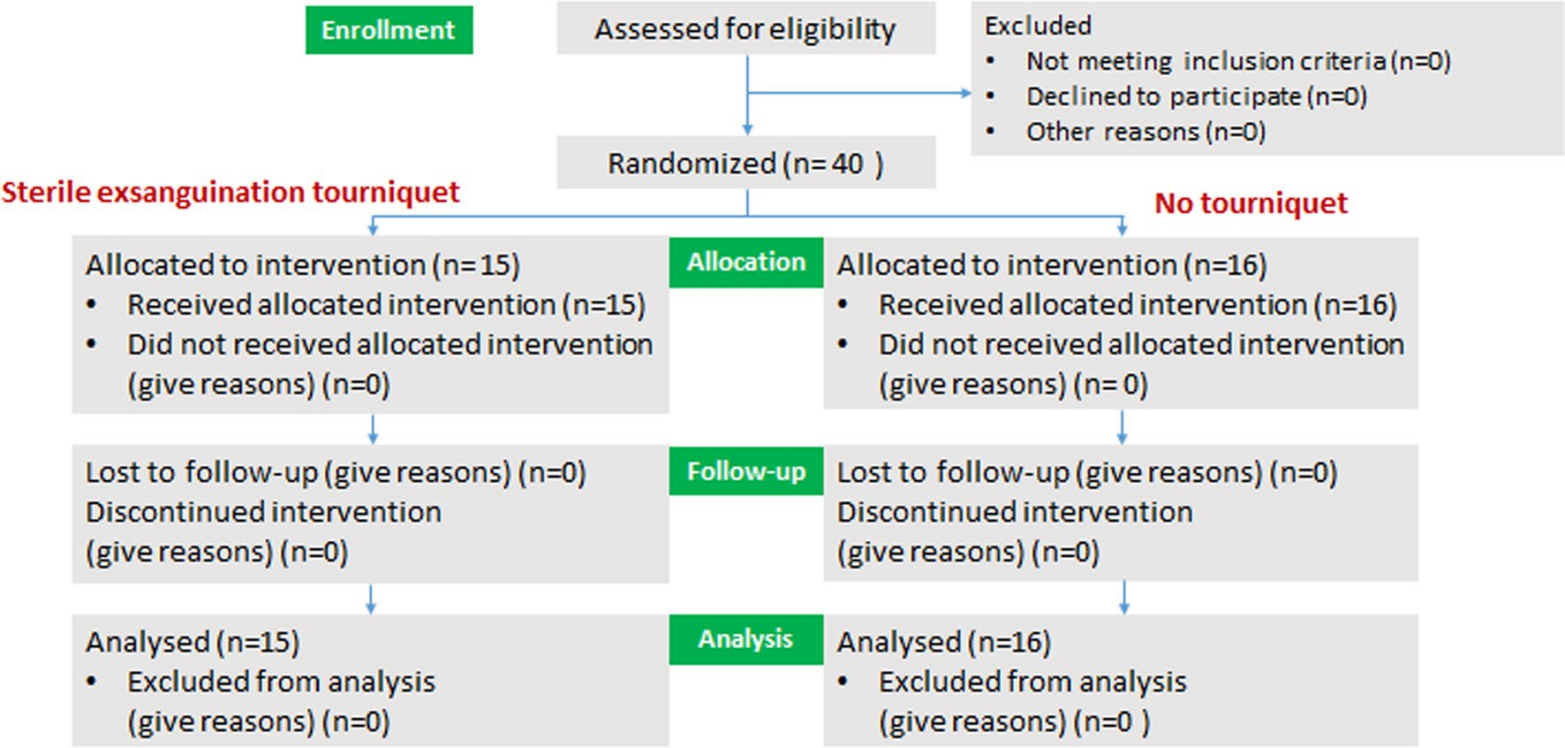
CONSORT diagram of patients enrolled in this study

Between September 2020 and September 2021, 31 eligible femoral-related procedures were enrolled in this study (Fig. 2). Of these procedures, 15 were randomized into the SET group and 16 into the control group. Table 1 summarizes the demographic data of patients between the two treatment arms. Femoral-related procedures, including fracture fixation, deformity correction, and bone lengthening, were equally distributed between the two treatment arms.

**Table 1:** Demographic data of patients between SET and the control group

Demographic data	SET (*n* = 15)	Control (*n* = 16)
	Mean ± SD	Mean ± SD
Age (years)	13.2 ± 4.0	11.4 ± 4.9
Gender (*n*, %)		
Male	9 (60.0%)	9 (56.3%)
Female	6 (40.0%)	7 (43.8%)
BMI (kg/m^2^)	20.9 ± 4.9	19.6 ± 5.2
Preoperative Hb (g/dl)	12.8 ± 1.4	13.0 ± 1.4
Femoral-related procedure (*n*, %)		
Fracture fixation	5 (33.3%)	5 (31.3%)
Deformity correction	8 (53.3%)	9 (56.3%)
Femoral lengthening	2 (13.3%)	2 (12.5%)

*SET* sterile exsanguination tourniquet

There was statistically significant lower body weight-adjusted TBL in the SET group (SET = 14.1 (7.7, 16.9) ml/kg vs. control 18.3 (14.8, 37.2) ml/kg, *p*-value = 0.027) (Table 2). Nevertheless, the interim analysis demonstrated that the body weight-adjusted transfusion volume was statistically significantly greater in the control group according to Pocock’s *p*-value adjustment (SET = 0.0 (0.0, 0.0) ml/kg vs. control = 2.1 (0.0, 9.7) ml/kg, *p* = 0.017). Accordingly, the recruitment for the study was prematurely halted due to the significant impact of transfusion volume observed in the control group.

**Table 2:** Primary and secondary outcomes between SET and the control group

Outcomes	SET (*n* = 15)	Control (*n* = 16)	*p*-value
	Median (ranges)	Median (ranges)	
*Primary outcomes*			
Body weight-adjusted total blood loss (ml/kg)	14.1 (7.7, 16.9)	18.3 (14.8, 37.2)	0.027
Body weight-adjusted transfusion volume (ml/kg)	0.0 (0.0, 0.0)	2.1 (0.0, 9.7)	0.017
*Secondary outcomes*			
Body weight-adjusted estimated intraoperative blood loss (ml/kg)	0.8 (0.2, 3.5)	5.6 (3.4, 21.5)	< 0.001
Body weight-adjusted estimated total drained volume (ml/kg)	2.4 (1.1, 8.6)	1.9 (1.0, 2.8)	0.100
24 h postoperative Hb (g/dl) (mean ± SD)	10.9 ± 1.8	10.5 ± 1.5	0.571
72 h postoperative Hb (g/dl) (mean ± SD)	10.7 ± 1.5	9.9 ± 1.5	0.172
Operative time (min)	105.0 (85.0, 125.0)	130.0 (101.3, 167.5)	0.039

*SET* sterile exsanguination tourniquet

Body weight-adjusted estimated intraoperative blood loss was significantly lower in the SET group (SET = 0.8 (0.2, 3.5) ml/kg vs. control = 5.6 (3.4, 21.5) ml/kg, *p* < 0.001). The total drained volume was not statistically significantly different between the SET group (2.4 (1.1, 8.6) ml/kg) and the control group (1.9 (1.0, 2.8) ml/kg) (*p* = 0.100). There were no differences in postoperative Hb at 24 h (*p* = 0.571) and 72 h (*p* = 0.172). In addition, the operative time was lower in the SET group with borderline statistical significance (SET = 105 (85.0, 125.0) vs. control = 130 (101.3, 167.5), *p* = 0.039).

## Discussion

This randomized trial evaluated the efficacy of SET in reducing perioperative blood loss and transfusion in pediatric femoral surgeries in which the standard pneumatic tourniquet cannot be used. An unplanned interim analysis revealed that SET significantly reduces the body weight-adjusted transfusion volume and estimated intraoperative blood loss without increasing the postoperative total drained volume. In addition, patients who underwent SET application during operation experienced less operative time with borderline statistical significance.

Several studies reported that SET was superior in exsanguination and could provide a bloodless surgical field more effective than the pneumatic tourniquet [5, 11, 12]. Brin et al. [13] study revealed that SET is more effective in minimizing postoperative hemoglobin reduction for total knee arthroplasty than a pneumatic tourniquet. Ladenheim et al. [11] reported a substantial reduction in perioperative blood loss for upper extremity hemodialysis vascular access procedures. Furthermore, SET is a single-use, sterile non-pneumatic tourniquet device, which is significantly associated with lower surgical contamination [14].

This study found that SET significantly reduced the body weight-adjusted transfusion volume estimated intraoperative blood loss for femoral-related orthopedic surgeries, similar to the previous study [5]. Unsurprisingly, patients in the control group were more likely to meet the blood transfusion criteria and were subjected to greater blood transfusion rates [15–17]. Our findings emphasize the benefit of SET in reducing the need for perioperative blood transfusion in femoral-related surgeries where the traditional pneumatic tourniquet is not applicable. In addition to the benefit of reducing perioperative blood transfusion, SET significantly reduced intraoperative blood loss and provided a bloodless surgery. As a result, better surgical field visualization could improve overall surgical competency resulting in an operative time reduction [4].

There were some limitations in this study. First, surgeon blinding was impossible, resulting in a potential for biases during the operation. Second, the interim analysis in this study was not prespecified. However, this interim analysis was recommended by an independent DSMB for patients’ safety. Nevertheless, the level of significance in this study has been adjusted for interim analysis.

## Conclusions

Utilization of a sterile exsanguination tourniquet (SET) significantly reduced a body weight-adjusted estimated intraoperative blood loss while preventing the need for blood transfusion within 24 h after pediatric orthopedic femoral-related surgeries. The reduced intraoperative blood loss allows surgeons to visualize the surgical field effectively, resulting in a shortened operative time. We suggest using SET for pediatric orthopedic femoral-related surgeries to maximize surgical competency, especially when the standard pneumatic tourniquet is inappropriate.
